# An Approach for Pulmonary Vascular Extraction from Chest CT Images

**DOI:** 10.1155/2019/9712970

**Published:** 2019-01-17

**Authors:** Wenjun Tan, Yue Yuan, Anning Chen, Lin Mao, Yuqian Ke, Xinhui Lv

**Affiliations:** ^1^College of Computer Science and Engineering, Northeastern University, Shenyang 110819, China; ^2^Cyberspace Institute of Advanced Technology, Guangzhou University, Guangzhou 510000, China

## Abstract

Pulmonary vascular extraction from chest CT images plays an important role in the diagnosis of lung disease. To improve the accuracy rate of pulmonary vascular segmentation, a new pulmonary vascular extraction approach is proposed in this study. First, the lung tissue is extracted from chest CT images by region-growing and maximum between-class variance methods. Then the holes of the extracted region are filled by morphological operations to obtain complete lung region. Second, the points of the pulmonary vascular of the middle slice of the chest CT images are extracted as the original seed points. Finally, the seed points are spread throughout the lung region based on the fast marching method to extract the pulmonary vascular in the gradient image. Results of pulmonary vascular extraction from chest CT image datasets provided by the introduced approach are presented and discussed. Based on the ground truth pixels and the resulting quality measures, it can be concluded that the average accuracy of this approach is about 90%. Extensive experiments demonstrate that the proposed method has achieved the best performance in pulmonary vascular extraction compared with other two widely used methods.

## 1. Introduction

At present, computed tomography (CT) has become the most common imaging modality for the diagnosis of lung disease. In analysis of chest CT scans, pulmonary vascular extraction is often required before proceeding to diagnose disease and also is an important step in the treatment planning, and follow-up of lung diseases [1]. In computer-aided diagnosis of lung disease, pulmonary vascular extraction can reduce ambiguities and improve nodule detection performance [2] and is used to aid detection of other pulmonary anatomical structures such as airway tree, pulmonary tissue, lung vein, and artery [3–5]. Due to the complexity of the anatomical structure of the pulmonary vascular and the influence of other anatomical structures having similar intensities (e.g., tumor nodules and dense lesion) [6], there is a still more complicated task of extract pulmonary vascular from large scale chest CT images, especially for the small vascular.

For the large amount of chest CT images, manual extraction is an extremely time-consuming and tedious task for doctors. The computerized semiautomatic and automatic methods may be helpful to reduce the doctor's effort. To address these issues, various methods have been extensively studied in recent years. Xu et al. [7] propose novel stagewise convolutional networks, followed by an orientation-based region-growing method, which learn discriminative features of pulmonary vessels automatically in a stage-by-stage manner by stagewise convolutional networks. Orkisz et al. [8] reported a vascular trees segmentation method by variational region growing. This process is performed within a lung mask, where the airways and bronchial walls were previously eliminated by adaptive multiscale morphological operations. Anna [9] introduced a 3D approach for segmentation of pulmonary vascular tree from CT thoracic scans based on author's experience in airway tree segmentation. Lai et al. [10] proposes an automatic integration segmentation approach for the vascular trees in low-dose CT scans. Zhu et al. [11] proposed a vascularity-oriented level set algorithm for pulmonary vessel segmentation in image-guided intervention therapy. Zhao et al. [12] proposed a vessel segmentation method is proposed for lung images based on a random forest classifier and sparse autoencoder features. Chen et al. [13] proposed a method to simultaneously and separately segment the pulmonary nodules and blood vessels. First, a line structure enhancement (LSE) filter and a blob-like structure enhancement (BSE) filter were used to augment the initial selection of vessel regions and nodule candidates, respectively. Then a front surface propagation (FSP) procedure was employed for precise segmentation of blood vessels and nodules. Buelow et al. [14] presented an automated method for the extraction of the pulmonary vessel tree from multislice CT data by a seed point–based front-propagation algorithm. Rudyanto et al. [15] presented an annotated reference dataset containing 20 CT scans and proposed nine categories to perform a comprehensive evaluation of vessel segmentation algorithms from both academia and industry. This dataset is used in the VESSEL12 challenge held at the International Symposium on Biomedical Imaging (ISBI) 2012. The currently most general and extensive vascular segmentation method can be found in [16], with a highly detailed categorization of the existing work. However, because the pulmonary vessel trees are very complex and have a huge number of branches, the overall number of works on pulmonary vascular extraction is very limited for the computer-assisted diagnosis of pulmonary disease and the major methods for pulmonary extraction are still to be developed [7, 17, 18].

Coming forward to meet these needs, a new approach for the extraction of pulmonary vascular from chest CT images is proposed in this work. The proposed approach is based on author's previous work in airway tree extraction [19]. The main idea of the proposed method is to extract the whole lung region, enhance the extracted lung region, and then extract the pulmonary vascular.

This paper is organized as follows. First, in Section 1 a brief review of existing approaches to segmentation of the pulmonary vascular from chest CT images is introduced.  Section 2 provides a detailed description of the proposed extraction approach. The image datasets, experiment software, and results of vascular extraction from chest CT image using the introduced method are presented and discussed in Section 3. Finally, Section 4 concludes the study.

## 2. Methods

**Figure 1:**
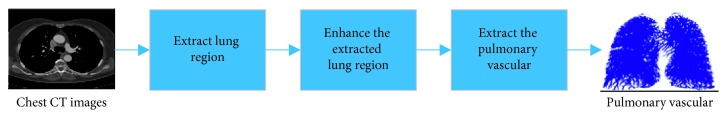
The flowchart of this method.

The method of this study mainly includes the following steps: extract lung region, enhance the extracted lung region, and extract the pulmonary vascular. The flowchart of the method in this study is shown in Figure 1. The specific algorithms for each step are described in the following sections.

### 2.1. Lung Region Extraction Method

**Figure 2:**
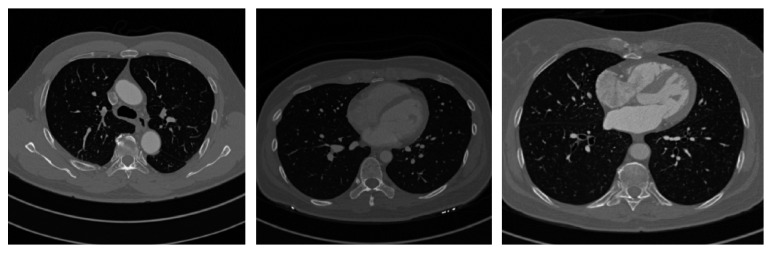
Chest CT images.

The anatomical structure of the lung in chest CT image is complex, as shown in Figure 2, if the pulmonary vascular is directly extracted in original CT images, it is difficult to remove the interference of other anatomical structures, such as bone, heart and muscle. Therefore, to segment accurately the pulmonary vascular, it is necessary to extract the lung region to remove other anatomical structures.

#### 2.1.1. Extract Lung Tissue with Region-Growing and Maximum Between-Class Variance Methods

The chest CT image is divided into two categories of lung tissue and other anatomical structure, in which grayscale values are significantly different in the CT image. Region-growing method can segment effectively and correctly a specific set of grayscale values in an image based on the grayscale difference between pixel of that image. The region-growing method is an iterative image segmentation method with three elementary parts: seed points selection, definition of similarity, and criteria for convergence to terminate the iterative process [20]. The regions are grown from these seeds to adjacent points depending on a region membership criterion (e.g., grayscale intensity). Keep examining whether the adjacent points of seeds should be classified into the seed points until the criterion is not met any more [21].

In the previous work, we have used the region-growing method to extract airway from chest CT images [19]. The extracted airway result showed that this method is simple and effective. Therefore, we also use the region-growing method to extract the lung tissue in CT images in this study. This method requires seed points of lung tissue from a particular slice image, which is the middle slice of the patient's chest CT image in this work.

First, the seed points are extracted by the method of maximum between-class variance, which can divide the original image into two parts by using threshold: foreground and background. In the chest CT image, the background is lung tissue and the foreground is the other anatomical structure. *T* is set to be the segmentation threshold of the foreground and background, the number of foreground points accounts for *ω*_0_ of the image, the average grayscale value is *u*_0_, the number of background points accounts for *ω*_1_ of the image, the average grayscale value is *u*_1_, and the total grayscale value of the image is as follows:

(1) $$\begin{array}{c} u=\omega_{0}u_{0}+\omega_{1}u_{1}. \end{array}$$

The variance of foreground and background images:

(2) $$\begin{array}{c} \sigma^{2}=w_{0}{\left(u_{0}-u\right)}^{2}+w_{1}{\left(u_{1}-u\right)}^{2}. \end{array}$$

Take u into formula (2) for calculating the variance of the two classes as follows:

(3) $$\begin{array}{c} \sigma^{2}=w_{0}w_{1}{\left(u_{0}-u_{1}\right)}^{2}. \end{array}$$

The maximum threshold *T*_max_ is obtained by using formula (3). The pixels of the image less than threshold *T*_max_ are extracted as the seed points of lung tissue.

Second, the similarity definition is used to determine whether the unmarked pixels of image are added to the detection region. This definition refers to the difference of image intensity between the adjacent pixels. The similarity condition is formulated [22] as follows:

(4) $$\begin{array}{c} \left|I\left(x_{k},y_{k}\right)-I\left(x,y\right)\right|<\theta ,\\ \left(x_{k},y_{k}\right)\in N_{26}. \end{array}$$

**Figure 3:**
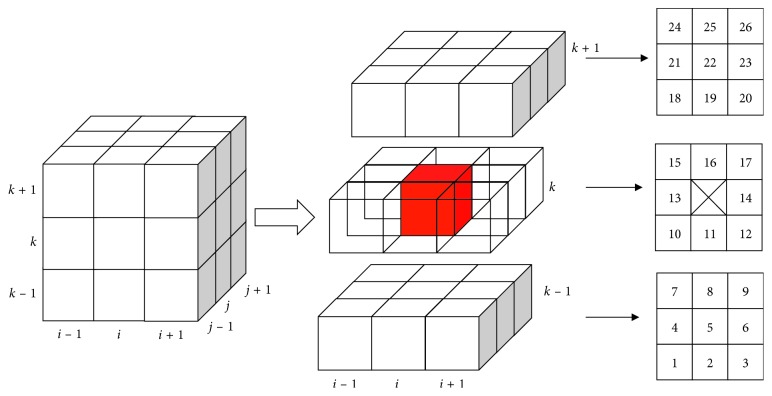
26-adjacent pixels of region growing.

The unmarked voxel (*x*_*k*_, *y*_*k*_) in the 26-adjacent *N*_26_ can be added to the lung tissue region, if the difference between the grayscale value of *I*(*x*_*k*_, *y*_*k*_) and seed voxel *I*(*x*, *y*) is less than the given threshold *θ*. And this voxel (*x*_*k*_, *y*_*k*_) is added to the seed queue voxel for next iteration. This process is shown in Figure 3.

Since this method relies on the difference between the grayscale value of the voxels in the 26-adjacent pixels, the given threshold *θ* plays an important role in this process. The extraction lung tissue regions are different with different given threshold. The threshold *θ* is calculated according to the following formula:

(5) $$\begin{array}{c} \theta =\frac{\max\;I_{\text{lung tissue}}\left(x,y\right)-\min\;I_{\text{lung tissue}}\left(x,y\right)}{2}, \end{array}$$

where max *I*_lung tissue_(*x*, *y*) and min *I*_lung tissue_(*x*, *y*) are the maximum and minimum gray value and of the pixels of the extracted seed points by maximum between-class variance method, respectively.

**Figure 4:**
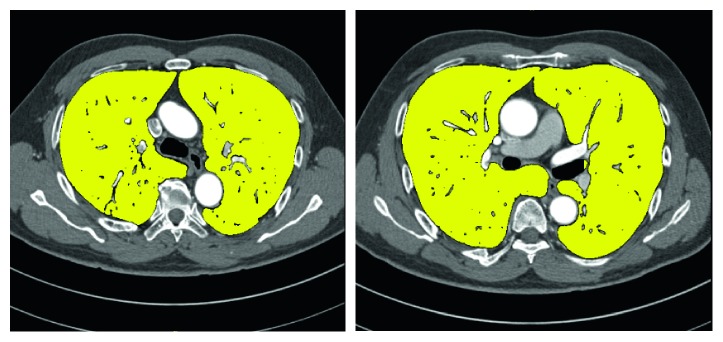
The extracted result of the lung tissue.

The extracted result of the lung tissue is shown in Figure 4, which shows that there are many holes in the extracted lung region. The reasons are that the grayscale value of the vascular is different from the lung tissue.

#### 2.1.2. Fill Holes of Extracted Lung Region with Morphological Operation

In this work, morphological operation method is used to fill the holes of lung tissue on the initial extraction region.

First, the contours of the lung are extracted in the all 2-D transverse images by 8-adjacent contour tracing method. There may be several contours in an image. The contours and the background are set to 1 and 0, respectively. And the set A is defined to represent the contours and the background pixels of the image. We formulate the filling “holes” function as

(6) $$\begin{array}{c} X_{k}=\left(X_{k-1}\;⊕\;B\right)\;\cap \;A^{c},\;k=1,2,3,\ldots , \end{array}$$

where *X*_0_=*p*, *p* is any point in the region, *B* is the 4-neighbourhood structural elements, ⊕ is the dilation operator, and *A*^*c*^ is the complement set of A. At the end of the iteration, *X*_*k*_=*X*_*k*−1_. The union set of A and *X*_*k*_ is the region filling the holes.

**Figure 5:**
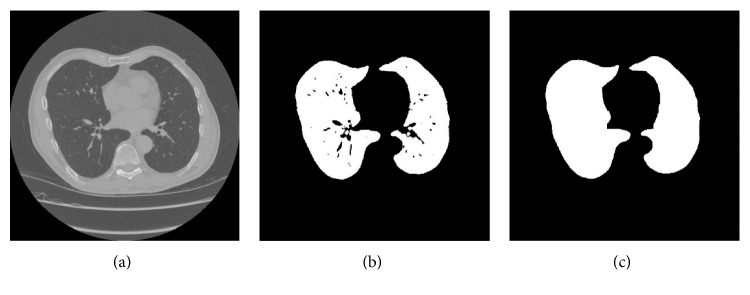
The final result of the extracted lung region: (a) original CT image, (b) lung tissue, and (c) the extracted lung region after filling holes.

The extracted region is expanded iteratively by the structural element. The number of iterations is manually based on experience. Then, the extracted region is corroded by the same structural element and the number of iterations of the expanding operation. The final result of the extracted lung region after filling holes is shown in Figure 5.

### 2.2. Vascular Enhancement Based on Fractional Differential

The actual chest CT image contains noise, which cause the edge of the pulmonary vascular of the image to be unclear. If the pulmonary vascular are directly extracted from the original CT image, it is easy to cause inaccurate extraction of small pulmonary vascular. Therefore, the fractional order differential operator is proposed for enhancing vascular region in the extracted lung tissue(as shown in Figure 4) region before extracting pulmonary vascular.

The Grümwald–Letnikov (G-L) is used to define the numerical implementation of fractional differential of image [23–25]. The period of the unary signal *w*(*t*) is *t* ∈ [*a*, *t*], and the signal period [*a*, *t*] is equally divided by a certain time interval (such as *h* = 1, 2, ...), then *n*=[(*t* − *v*)/*h*]^*h*=1^=[*t* − *v*], and thus, the differential expressions with fractional differentials can be obtained:

(7) $$\begin{array}{c} \frac{d^{v}w\left(t\right)}{dt^{v}}\approx w\left(t\right)+\left(-v\right)w\left(t-1\right)+\frac{-v\left(-v+1\right)}{2}w\left(t-2\right)+\ldots +\frac{\Gamma \left(-v+1\right)}{n!\Gamma \left(-v+n+1\right)}w\left(t-n\right). \end{array}$$

For the CT image, the differential expressions with fractional differentials can be obtained as follows:

(8) $$\begin{array}{c} \frac{\partial^{v}w\left(x,y\right)}{\partial x^{v}}\approx w\left(x,y\right)+\left(-v\right)w\left(x-1,y\right)+\frac{-v\left(-v+1\right)}{2}w\left(x-2,y\right)+\ldots +\frac{\Gamma \left(-v+1\right)}{\left(n-1\right)!\Gamma \left(-v+n\right)}w\left(x-n+1,y\right), \end{array}$$

(9) $$\begin{array}{c} \frac{\partial^{v}w\left(x,y\right)}{\partial y^{v}}\approx w\left(x,y\right)+\left(-v\right)w\left(x,y-1\right)+\frac{-v\left(-v+1\right)}{2}w\left(x,y-2\right)+\ldots +\frac{\Gamma \left(-v+1\right)}{\left(n-1\right)!\Gamma \left(-v+n\right)}w\left(x,y-n\right). \end{array}$$

The above differential expression constructs 3 *∗* 3 or 5 *∗* 5 differential operators to process the image to enhance the edge of the pulmonary vascular. Since the differential operator is isotropic, it does not enhance the small pulmonary vascular information with the characteristic direction. Thus, a fractional differential operator template can be constructed according to formulae (8) and (9). The operator has the following effect: it has little effect on the smooth region, where the surrounding gray value changes little; and the operator plays an enhancement role for the pixels whose surrounding gray value changes greatly.

**Figure 6:**
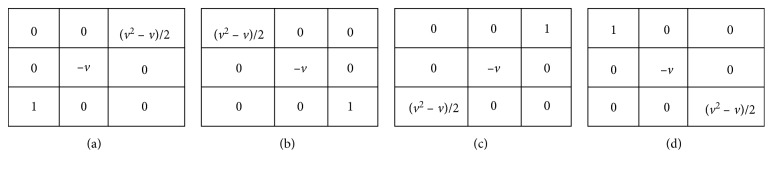
3 *∗* 3 fractional differential operator template.

By analysing the characteristics and distribution of pulmonary vascular in the chest CT images, it is known that the pulmonary vascular are cylindrical and their directions are mostly four diagonal directions. Thus, a 3 × 3–differential operator template is constructed to enhance the region of vascular, as shown in Figure 6.

Since the operator is not symmetric, the following method should be used: the coordinates of the “1” of the operator template should coincide with the coordinates of the pixel to be subjected to the fractional differential operation. The templates in these four directions are convolved with the lung tissue region, and then the convolution results in each direction are weighted, and the results in the four directions are summed. The image after pulmonary vascular enhancement can be expressed by formula (10):

(10) $$\begin{array}{c} g\left(x,y\right)=4w\left(x,y\right)-v\left(w\left(x+1,y-1\right)+w\left(x-1,y+1\right)+w\left(x-1,y-1\right)+w\left(x+1,y+1\right)\right)+\left(v^{2}-v\right)\left(w\left(x+2,y-2\right)+w\left(x-2,y+2\right)+w\left(x-2,y-2\right)+w\left(x+2,y+2\right)\right). \end{array}$$

**Figure 7:**
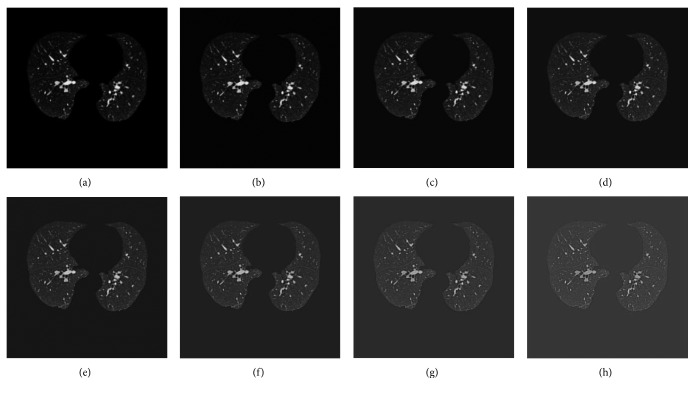
The result of different differential order: (a) original CT image, (b) 0.1 order, (c) 0.2 order, (d) 0.3 order, (e) 0.4 order, (f) 0.5 order, (g) 0.6 order, (h) 0.7 order.

The lung region is processed using the above 3 *∗* 3 fractional differential enhancement template. The different order can obtain different enhancement results. Therefore, we first study the experimental results of different fractional orders for chest CT image, which is shown in Figure 7. The results show that the pulmonary vascular in the CT image are enhanced as the differential order increases. At the same time, the result of small pulmonary vascular enhancement between 0.1 and 0.2 is the best. Although the pulmonary vascular details are enhanced when the order is greater than 0.2, large pulmonary vascular are also suppressed, and the contrast between the large pulmonary vascular and other lung tissues is lowered. Thus, 0.2 fractional order is used to enhance the lung region of CT image in this work.

### 2.3. Extract Pulmonary Vascular by FMM

The complete lung region is extracted by the method of Section 2.1 and enhanced by fractional differential method. In this section, the pulmonary vascular is extracted with the fast marching method in the lung tissue region.

The fast marching method originates from the solution of the equation of the distance function. Suppose there is a curve *φ* moving in one direction. The FMM can be described as a family of schemes for computing the evolution of fronts. Things become interesting when the front evolves over time. In the context of the fast marching method, the speed does not have to be the same everywhere but the speed must always be non-negative. As a given point, the motion of the front is described by the equation known as the Eikonal equation, which can be expressed as [13]:

(11) $$\begin{array}{c} \left\|\nabla T\left(x\right)\right\|F\left(x\right)=1,\\ T\left(\varphi \right)=0, \end{array}$$

where *T*(*x*) is the arrival time of the front at point *x* and *F*(*x*) ≥ 0 is the speed of the front at point *x*.

This is the general form of the equation of distance function. Godunov [26] gives a solution:

(12) $$\begin{array}{c} \max{\left(D_{ij}^{-x}T,-D_{ij}^{+x}T,0\right)}^{2}+\max{\left(D_{ij}^{-y}T,-D_{ij}^{+y}T,0\right)}^{2}=\frac{1}{V_{ij}^{2}}. \end{array}$$

In this solution, *D*_*ij*_^−^ and *D*_*ij*_^+^ are backward difference and forward difference operators, *i* and *j* represent the two adjacent points of the curve *φ*, respectively.

If ∇T is approximated as a first-order finite difference operator, then formula (12) can be written as

(13) $$\begin{array}{c} \overset{2}{\underset{v=1}{\sum }}\max{\left(\frac{T-T_{v}}{\Delta v},0\right)}^{2}=\frac{1}{V^{2}}. \end{array}$$

In that case, Δ_1_=Δ_*x*_, Δ_2_=Δ_*y*_, *T*=*T*_*ij*_, *V*=*V*_*ij*_, *T*_1_=min(*T*_*i*+1,*j*_, *T*_*i*−1,*j*_), *T*_2_=min(*T*_*i*,*j*+1_, *T*_*i*,*j*−1_).

A distance value for image must be found to estimate the length of the gradient ‖∇*T*‖ is equal to 1/*F*. The following formula (13) is proposed to solve ‖∇*T*‖:

(14) $$\begin{array}{c} \left\|\nabla T\right\|={\left(D_{i,j}^{2}+D_{i,k}^{2}+D_{j,k}^{2}\right)}^{1/2}. \end{array}$$

The *D*_*i*,*j*_, *D*_*i*,*k*_, *D*_*j*,*k*_ represent the gradient value in the three directions of Cartesian coordinate system, respectively. *i*, *j*, and *k* represent the three adjacent points.

**Figure 8:**
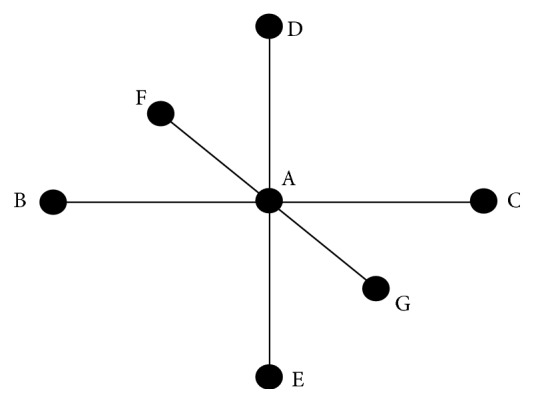
Adjacent points of three-dimensional CT images.

Considering the case of adjacent points of three-dimensional CT images (as shown in Figure 8), formula (14) can be written as follows:

(15) $$\begin{array}{c} \left\|\nabla T\right\|={\left(\max{\left(V_{A}-V_{B},V_{A}-V_{C},0\right)}^{2}+\max{\left(V_{A}-V_{D},V_{A}-V_{E},0\right)}^{2}+\max{\left(V_{A}-V_{F},V_{A}-V_{G},0\right)}^{2}\right)}^{1/2}, \end{array}$$

where *V*_*A*_ is the unknown distance value and *V*_*B*_, *V*_*C*_, *V*_*D*_, *V*_*E*_, *V*_*F*_, *V*_*G*_ are the distance values at the neighbouring voxels. The distance value is the gray value of the pixels of CT images.

For speeding up in homogeneous regions of the image and slowly in regions with a high value of image gradient. The *F* of the chest CT volume data, be considered as gradient figure, is computed by the exponentiated gradient algorithm as follows:

(16) $$\begin{array}{c} F=e^{-k\left\|\nabla T\right\|}. \end{array}$$

The solution process of FMM is described as follows:

**Step 1 .**

All the points are categorized into three categories: the processing points are the seed points (in the initial step, the number of the seed points is one; during the processing, the seed points may be many points); the boundary points are the points near the processing points; the pending points are all the remaining points.

**Step 2 .**

The arrival time *T*(*x*) is calculated from the initial seed point to the boundary points and sort them according to the time from small to large.

**Step 3 .**

The boundary points, less than a certain arrival time threshold, are marked as the next processing points of as the next processing points. Other boundary points are marked as the background points. The nearby points of the next processing points are marked as the new boundary points.

**Step 4 .**

The arrival time *T*(*x*) of new boundary points are calculated and sorted. If the next processing points are empty, the processing is finished. Otherwise, it returns to (2).

The above processing can be considered that the curve spread along an equal timeline until it arrives at the front of a wall.

In this work, the full lung region of the chest CT images is extracted by the method of Section 2.1 and enhanced by fractional differential method. Then the points of pulmonary vascular are extracted with the maximum between-class variance method in the full lung region of the middle of slice of the chest CT images. In this case, the foreground is the pulmonary vascular. These extracted points are selected as initial seed points. And the minimum and maximum grayscale values of these pulmonary vascular points are calculated as the limited grayscale value threshold. According to the limited grayscale value threshold and the gradient field figure F, the seed points begin to spread through the lung region of images using the fast march method as the above solution process. The points of pulmonary vascular are extracted completely when the spread processing stops.

## 3. Results and Discussion

The 3 chest CT images dataset were used in this experiment with resolution of 512 × 512, slice thickness less than 1.5 mm and slice number more than 350, which are from a hospital. In order to protect patient privacy information, the image dataset hides the hospital's and patient's name. The ground truth is manually drawn by the doctor for each database.

This method was implemented in Matlab 2015 on a PC with 4 Intel© Core is-i7-6700U CPUs 2.60 GHz, 8 GB DDR4 RAM and NVIDIA GeForce 940 Mx GPU with 2 GB video memory.

We will quantitatively analyse the results of the method for extraction of pulmonary vascular. By comparing the extraction results of the proposed method with the ground truth image, the accurate rate, the leakage rate and over rate of the proposed method are calculated to quantitatively evaluate the. The ground truth image, be manually drawn by the professional doctors on the original CT image, is provided in VESSEL12 challenge.

The evaluation criteria for pulmonary vascular segmentation are as follows:

(17) $$\begin{array}{c} \text{accuracy}=\;\frac{S\;\cap \;T}{T},\\ \text{leakage rate}=\frac{T-S\;\cap \;T}{T},\\ \text{over rate}=\frac{S-S\;\cap \;T}{T}, \end{array}$$

where *T* and *S* is the number of pulmonary vascular pixels in the ground truth image and the extraction result by this method, respectively.

**Figure 9:**
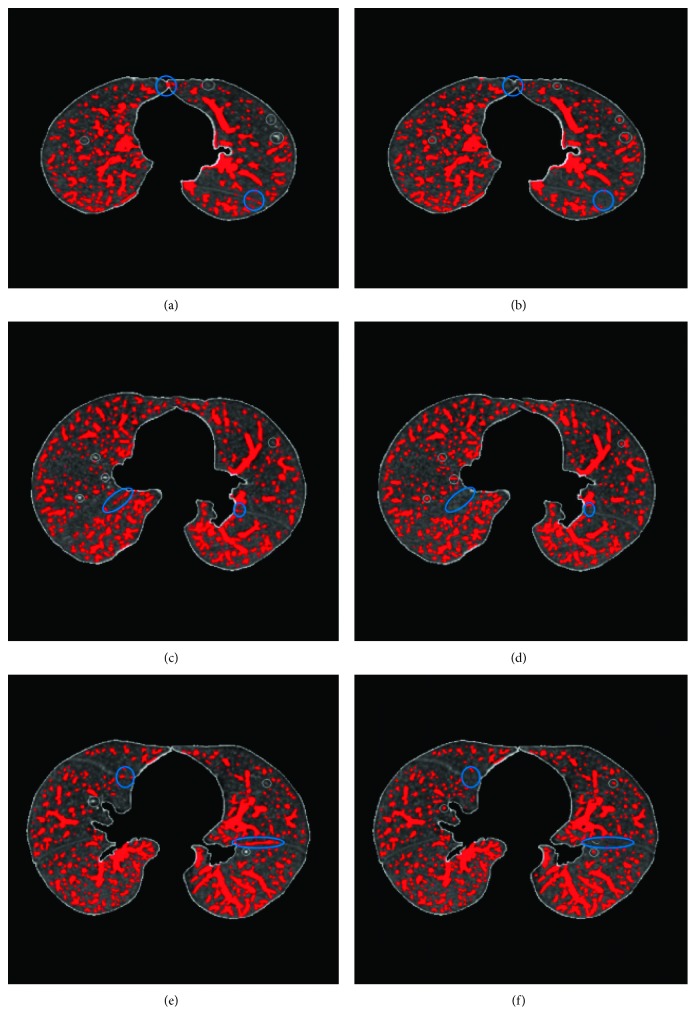
The comparison results between this method and the ground truth: (a–c) are the results of this method and (b–f) are corresponding ground truth.

In order to observe the experimental results, the comparison results of the 3 datasets between the method in this paper and the ground truth is shown in Figure 9. Figures 9(a) and 9(c) © are the extraction results of this method, and Figures 9(b), 9(d), and 9(f) are the ground truth results. The blue circles indicate different places between the method and the ground truth. The results show that the method can completely extracted the pulmonary vascular, even the extraction results of small pulmonary vascular are also very good, and cause a little over segmentation in the edge of lung region.

The method is compared with the level set [11] and regional growth algorithm [8], which are widely used to extract pulmonary vascular from medical image. The accurate rate, the leakage rate, and the over rate of each segmentation method are calculated by comparing with the ground truth image.

**Figure 10:**
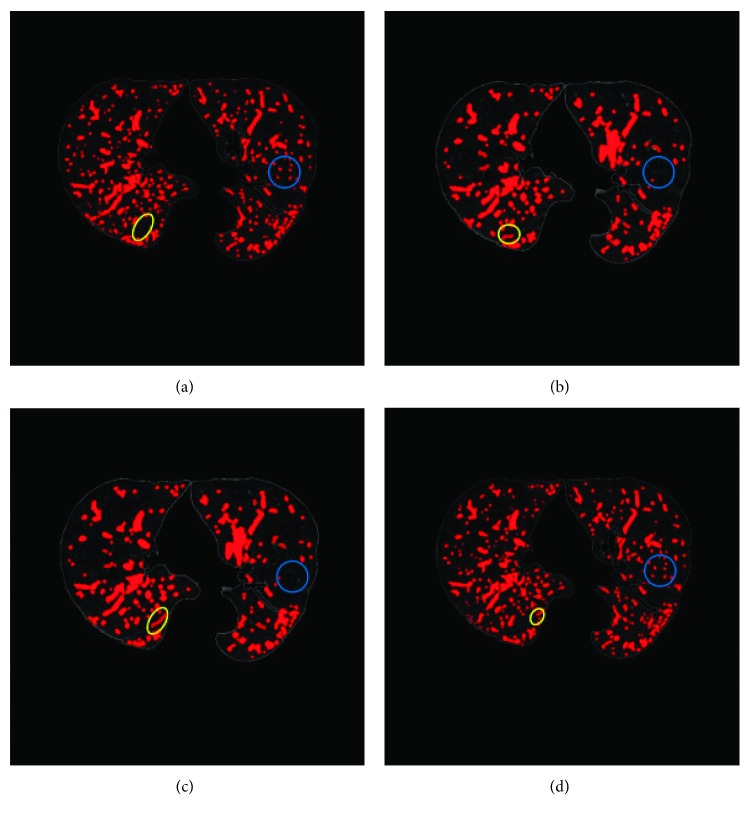
Compare the results of different segmentation methods and the ground truth: (a) ground truth, (b) result of level set method, (c) result of region-growing method, and (d) result of this method.

The experimental results of an image dataset of different segmentation methods are shown in Figure 10. The different places of these results are shown by yellow and blue circles in the result images. The results show that the segmentation results of these four methods are equally good, but this method is a better accurate segmentation of the small pulmonary vascular and less over segmentation than the other methods. The blue circle of Figure 10 shows that this method can extract more the small pulmonary vascular. Meanwhile, the yellow circle of Figure 10 shows that this method is less over segmentation results than the other methods.

**Figure 11:**
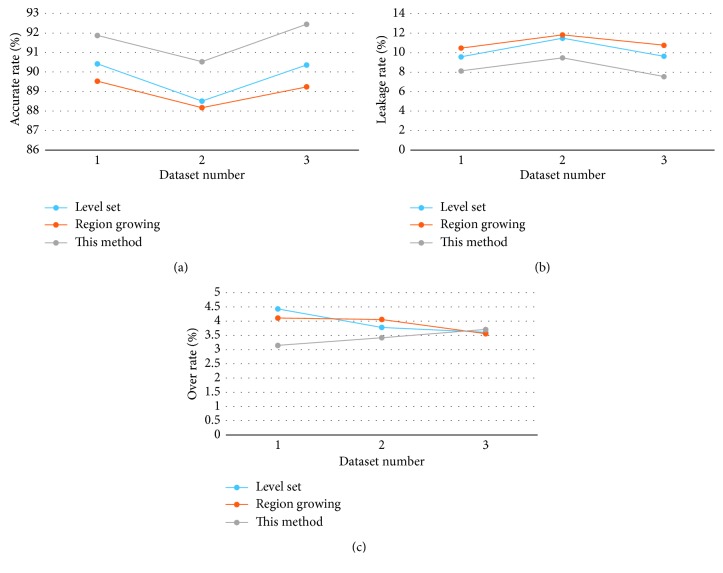
Comparison of segmentation results of three methods: (a) accurate rate, (b) leakage rate, and (c) over rate.

Table 1 and Figure 11 are the statistical analysis of the segmentation results of the three methods. The average of accurate rate of this method is 91.62%, which is better than the other two methods. And the average of leakage rate and over rate of this method is 8.38% and 3.43%, respectively. It is less than the other two methods.

In general, the running time of segmentation method is related to the slice thickness and the slice number. The average running time of this method is about 2 minutes, which can meet the requirements of clinical real-time operation. Furthermore, this method is completely automatic segmentation process without manual operation.

## 4. Conclusions

This study proposes a method for segmenting pulmonary vascular from chest CT images. The lung tissue region is extracted from chest CT images by region-growing method and maximum between-class variance. Then, the holes of the extracted region are filled by morphological operation. The points of the pulmonary vascular of the middle slice of the CT images are extracted as the original seed points. Finally, the seed points are spread throughout the lung tissue based on the fast-marching method to extracted the pulmonary vascular with the gradient image. The experimental results show that this method can completely extracted the big and small pulmonary vascular and the accuracy is better than the level set method and region-growing method, and the leakage rate and over rate of this method is less than the two other methods. Furthermore, the running time of this method is about 2 minutes to meet the requirements of clinical real-time operation. This method may be used to promote the diagnosis technology of lung diseases for clinical application.

## Figures and Tables

**Table 1:** The statistical analysis of the segmentation results of the three methods.

Dataset no.	Level method (%)			Region-growing method (%)			This method (%)		
	Accurate rate	Leakage rate	Over rate	Accurate rate	Leakage rate	Over rate	Accurate rate	Leakage rate	Over rate
1	90.42	9.58	4.43	89.53	10.47	4.11	91.87	8.13	3.15
2	88.51	11.49	3.78	88.17	11.83	4.06	90.53	9.47	3.42
3	90.36	9.64	3.61	89.24	10.76	3.56	92.45	7.55	3.71
Average	89.76	10.24	3.94	88.98	11.02	3.91	91.62	8.38	3.43

## Data Availability

The data used to support the findings of this study are available from the corresponding author upon request.
